# Medical and Physical Intelligence

**Published:** 1806-04

**Authors:** 


### MEDICAL AND PHYSICAL INTELLIGENCE.

A letter from Ragusa, inserted in the Vienna Court Gazette, says, "The vaccine inoculation has at length triumphed here through the zeal and the efforts of the indefatigable Dr. Stulli, who, at the repeated invitations of Dr. Carro of Vienna, has happily surmounted all the obstacles which prejudice and carelessness threw in his way. The Catechism written by Dr. Carro, being translated into the Illyrian language, and circulated in the town and adjacent country, induced a great number of the inhabitants to adopt inoculation. The matter transmitted from Vienna produced the best effect. In a few days Dr. Stulli inoculated 100 children, which is a considerable number for this country, in which of late years, and even in 1804, more than 300 children died of the natural small-pox.

---

Dr. Richter has lately discovered a metal which resembles, in many respects, nickel, and which he calls Niccolanum. In comparing this substance with cobalt and nickel, we are told it resembles, 1. by its property of surcharging itself with oxygen at the expence of the nitric acid, and thus forming a body which resembles the black oxyd of manganese, with respect to its solubility in the acids; 2. by its property of not being reducible without the intervention of a combustible body. It differs from cobalt, 1. by the blackish green colour of its solutions, even when they are neutralized; 2. by the colour of its carbonate; that of cobalt is of a beautiful purple, but that of niccolanum is of a blueish green, inclining to a pale grey; 3. by the colour of its oxyd precipitated without carbonic acid; that of cobalt is of a deep blue, and changes, even during washing, into a blackish brown, but the oxyd of niccolanum is of a bluish green. Niccolanum resembles nickel, 1. by its strong magnetic quality; 2. by its malleability; 3. by the deep green colour of its solutions; 4. by the loss of this colour when its neutral combinations are deprived of water; 5. by the colour of its acid solution with an excess of ammoniac. It differs from nickel, 1. because it cannot be reduced without the intervention of a combustible body; 2. because nitric acid attacks and oxydates it more easily; 3. by the colour of its combinations, and by the colour of its precipitates. M. Che-

M. Chevalier, of Berlin, has invented a new varniſh applicable to wood, plaster, and other ſubſtances, and which reſiſts the action of water, air, and alkaline leys. It is thought that wood, employed for the conſtruction of ſhips or houſes, or for piles ſunk in water or wet earth, coated over with this varniſh, will endure three times as long, and run no riſk of rotting. Two pounds and a half of the varniſh are ſufficient for a ſurface of a hundred ſquare feet, and is ſold at eighty-two Pruſſian dollars the Cwt.

---

Mr. Hawker, of Dudbridge, Glouceſterſhire, has lately exhibited, at Sir Joſeph Banks's, complete drawings, and ſeveral of the bones of a large foſſile animal, ſimilar to the crocodile, found in a ſolid ſtratum of lime-ſtone twenty feet thick. It was imbedded fifteen feet below the ſurface of the ſtratum. The ſkeleton meaſures 10 ½ feet in length, and all the parts are wonderfully perfect. The jaws contained the teeth in high preſervation, and ſtill covered with enamel. One of them, which was broken, had ſo exactly the fracture of what is called petrified wood, as to furniſh a ſtrong ground for ſuſpicion that many foſſils, generally held to be of vegetable, are of animal origin. In the ſame ſtratum of lime-ſtone are found many cornua ammonis, muſcles, and other ſhells.

---

In February a letter was read at a meeting of the Royal Society, from Mr. Griffiths to the Preſident, containing a brief account of a ſpecies of worm-ſhell, found by him in a bank of clay, on the coaſt of Sumatra, after the ſhock of an earthquake. Conſiderable numbers of the ſame ſpecies are found in the ſurrounding ſeas, in water from one to ſix fathoms deep; they vary in length from three to five feet, and in diameter from three to nine inches. One of the ſpecimens procured by Mr. Griffiths, meaſures above five feet, is taper, has two tentaculi, and in other reſpects, is ſomewhat different from the figure given by Rhumphius. Not one of the ſhells could be procured perfect in itſelf; but their true figure may be correctly conceived from the different broken ſpecimens. The body of the fiſh conſiſted of a gelatinous maſs. The outſide of the ſhell was white, the inſide yellow, and its fracture ſtrongly reſembled ſtalactite.

---

Some experiments have lately been made at Amiens, to aſcertain the nature of the foul air in oil ciſterns; and it has been found to conſiſt of azotic gas, oxygenated gas, and carbonic acid gas, in the proportions of 86, 8 and 6.

---

A work upon the cauſe and treatment of the gout, by the late Dr. Hamilton, of Lynn, Author of Obſervations on Scrophulous Affections, Marſh Remittent Fever, &c. is in the preſs. This work contains much new matter upon the nature and origin of that verſatile diſeaſe, the gout, and a ſucceſsful mode of treating it, experienced in the author's own perſon.

---

Mr.

Mr. Creaser, Surgeon of Bath, has nearly ready for publication, ſome extenſive Reports on the Medical Application of Galvaniſm.

Mr. Blair has a work in the preſs, nearly ready for publication, in which he makes the perſons who affect to rail at the Cow-pox refute themſelves, by quoting their own words in a dialogue.

Mr. Gunning, Surgeon to St. George's Hoſpital, propoſes to give, in October next, at his houſe in Clifford Street, A Courſe of Lectures on the Principles and Operations of Surgery, illuſtrated by a Reference to the Caſes which occur at St. George's Hoſpital. For farther particulars, apply to Mr. Gunning, No. 1, Clifford Street, Bond Street.

Mr. Thelwall has opened a Seminary, No. 40, Bedford Place, Ruſſell Square, for the cultivation of the Science and Practice of Elocution, and the Cure of Impediments of Speech; and is delivering a Courſe of Lectures on the Elementary Principles of his Art. The Lectures are illuſtrated by graphic and mechanical demonſtrations of the eſſential Propoſitions; and are occaſionally relieved by popular readings and recitations, and ſpecimens of ſpontaneous oratory. Mr. T. alſo propoſes to receive into his houſe a limitted number of pupils who have impediments of utterance, of whatever deſcription, the deaf alone excepted; and to give leſſons, at ſtated hours, to individuals and to ſelect claſſes, to whom domeſtication might not be convenient; and who may either labour under the like imperfections, or be deſirous of improvement in the accompliſhments of reading and recitation, converſational fluency, public oratory, and the principles of criticiſm and compoſition.

